# Whole genome sequencing and protein structure analyses of target genes for the detection of *Salmonella*

**DOI:** 10.1038/s41598-021-00224-7

**Published:** 2021-10-22

**Authors:** Lijun Hu, Guojie Cao, Eric W. Brown, Marc W. Allard, Li M. Ma, Guodong Zhang

**Affiliations:** 1 Division of Microbiology, Office of Regulatory Science, Center for Food Safety and Nutrition, U.S. Food and Drug Administration, 5001 Campus Dr., College Park, MD 20740 USA; 2 Institute for Biosecurity and Microbial Forensics, Department of Entomology and Plant Pathology, Oklahoma State University, Stillwater, OK 74074 USA

**Keywords:** Bacteria, Pathogens

## Abstract

Rapid and sensitive detection of *Salmonella* is a critical step in routine food quality control, outbreak investigation, and food recalls. Although various genes have been the targets in the design of rapid molecular detection methods for *Salmonella*, there is limited information on the diversity of these target genes at the level of DNA sequence and the encoded protein structures. In this study, we investigated the diversity of ten target genes (*inv*A, *fim*A, *pho*P, *spv*C, and *agf*A; *ttr*RSBCA operon including 5 genes) commonly used in the detection and identification of *Salmonella*. To this end, we performed whole genome sequencing of 143 isolates of *Salmonella* serotypes (Enteritidis*,* Typhimurium*,* and Heidelberg) obtained from poultry (eggs and chicken). Phylogenetic analysis showed that *Salmonella* ser. Typhimurium was more diverse than either Enteritidis or Heidelberg. Forty-five non-synonymous mutations were identified in the target genes from the 143 isolates, with the two most common mutations as T ↔ C (15 times) and A ↔ G (13 times). The gene *spv*C was primarily present in *Salmonella* ser. Enteritidis isolates and absent from Heidelberg isolates, whereas *ttr*R was more conserved (0 non-synonymous mutations) than *ttr*S, *ttr*B, *ttr*C, and *ttr*A (7, 2, 2, and 7 non-synonymous mutations, respectively). Notably, we found one non-synonymous mutation (*fim*A-Mut.6) across all *Salmonella* ser. Enteritidis and *Salmonella* ser. Heidelberg, C → T (496 nt postion), resulting in the change at AA 166 position, Glutamine (Q) → Stop condon (TAG), suggesting that the *fim*A gene has questionable sites as a target for detection. Using Phyre^2^ and SWISS-MODEL software, we predicted the structures of the proteins encoded by some of the target genes, illustrating the positions of these non-synonymous mutations that mainly located on the α-helix and β-sheet which are key elements for maintaining the conformation of proteins. These results will facilitate the development of sensitive molecular detection methods for *Salmonella.*

## Introduction

Foodborne diseases caused by *Salmonella* are an ongoing public health problem and a global economic burden. Non-typhoidal *Salmonella* caused an estimated 1,027,561 illnesses, 19,336 hospitalizations, and 378 deaths annually in the United States^1^. In 2010, World Health Organization (WHO) reported that non-typhoidal *Salmonella enterica* was the top causing agent responsible for 59,000 of the 230,000 (25.65%) deaths, and 4 million of the 18 million (22.22%) Disability Adjusted Life Years (DALYs) attributed to diarrheal disease agents^2^. *Salmonella-*contaminated foods, especially poultry-derived foods (eggs, chicken meat), are the most significant source of salmonellosis^3^. *Salmonella* serotypes Enteritidis*,* Typhimurium*,* and Heidelberg are consistently reported as the most frequent *Salmonella* serotypes associated with egg and poultry products^4–6^. However, global egg and chicken consumptions have been rising steadily; egg and chicken meat production increased from 14.7 to 66.4 million tons (350.2%) and 7.9 to 92.8 million tons (1077.2%), respectively, from 1962 to 2012^7^. Therefore, the routine surveillance, detection, and prevention of *Salmonella* in poultry products are extremely important to public health.

Detection methods are essential for surveillance and prevention of foodborne pathogens. All effective molecular detection methods (such as PCR based methods and isothermal methods) require target genes. The target genes *inv*A, *ttr*RSBCA (operon including 5 genes of *ttr*R, *ttr*S, *ttr*B, *ttr*C, *ttr*A), *pho*P, *fim*A, *agf*A, and *spv*C have been frequently used either separately or in combination for *Salmonella* detection in many PCR and loop-mediated isothermal amplification (LAMP) assays^8–18^. All these target genes are located on chromosome, except for *spv*C, which is encoded on a plasmid. The proteins they encode have a variety of functions and are all involved in virulence, such as participation in the biosynthesis of flagella via an encoded putative inner membrane protein (*inv*A), tetrathionate respiration (*ttr*RSBCA operon), regulation and two-component signaling (*pho*P), adherence and fimbriae activity (*fim*A and *agf*A), and promotion of the survival and rapid growth of *Salmonella* in the host (*spv*C). Considering the importance of these genes in the development of molecular detection methods for *Salmonella*, variations in their sequences among *Salmonella* isolates may impact the target coverages of these detection methods. The recent advances in whole-genome sequencing (WGS) technology and bioinformatics provide powerful tools for studying the possible variations in these target genes effectively among large number of *Salmonella* isolates. Furthermore, WGS and bioinformatics are also the most powerful tools to genotype various microorganisms, including *Salmonella*. There are WGSs from 155,509 *Salmonella enterica* isolates provided online^20^ and these data have been widely used to track and investigate outbreaks. For example, WGS was used by Inns and colleagues^21^ to investigate 136 *Salmonella* ser. Enteritidis cases from an outbreak in the United Kingdom in 2015, including 21 travel-associated cases of salmonellosis. That outbreak was traced back to chicken eggs and further linked to 18 contemporaneous cases reported in Spain (18 identified cases). In another similar study, WGS data and food trace-back investigations identified that eggs used at food premises were the sources of *Salmonella* ser. Typhimurium contamination in seven outbreaks in Australia, with WGS technology providing a higher discriminatory ability than multiple-locus variable-number tandem repeat analysis (MLVA)^22^.

As serotypes of Enteritidis, Typhimurium, and Heidelberg are the common causes of salmonellosis worldwide, we chose to study a group of representative isolates of these serotypes sourced from poultry. The purposes of this study were to (1) determine the sequence variation of target genes (*inv*A, *ttr*RSBCA, *fim*A, *pho*P, *spv*C, and *agf*A) among these isolates; (2) predict the protein structures of the selected genes to identify the potential impact of the mutations on protein functions; and (3) provide a detailed comparative genomic analysis of these major serotypes. The study adds additional WGS data and new protein structure information to the *Salmonella* online database and provide insights for the improvement in the design of rapid molecular detection methods for *Salmonella*.

## Results

### WGS and Phylogenetic analyses of the three *Salmonella* serotypes

The overall results of WGS, such as the number of assembled bases and N50 contig sizes, are summarized in Supplementary Tables 1–3 and Supplementary Fig. 1. In general, *Salmonella* genomes averaged at the size of approximately 5 Mb, the number of contigs ranged from 28 to 473, and the average depth of coverage ranged from 25 × to 433x (Supplementary Tables 1–3). The WGS data of all the isolates studied here can be accessed from the NCBI SRA (https://www.ncbi.nlm.nih.gov/sra) with their accession numbers listed in Supplementary Tables 1–3.

**Figure 1:**
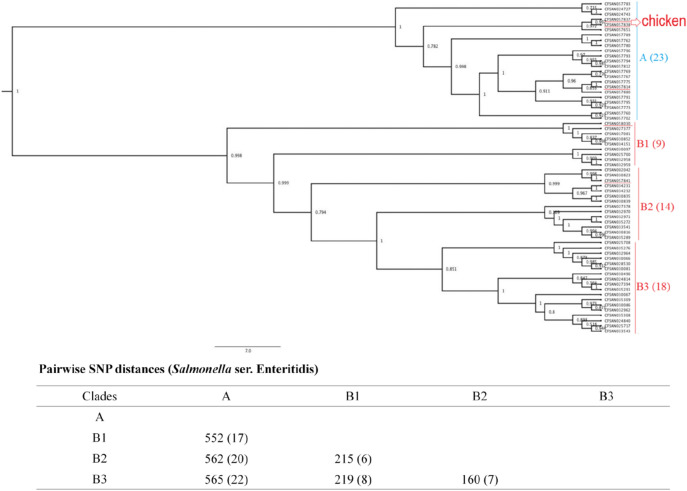
Phylogenetic tree of 64 *Salmonella* ser. Enteritidis isolates. Using core SNPs determined by FDA CFSAN SNP pipeline, the tree was constructed using the GTR-CAI model of FASTree 2. The analysis involved 552–565 total SNP positions. Clades and subclades are indicated in colors (**A**, blue; and **B**, red) with numbers of total isolates in each clade or subclade in parentheses. Isolates sourced from chicken samples were highlighted under red line, the rest were from egg samples.

**Figure 2:**
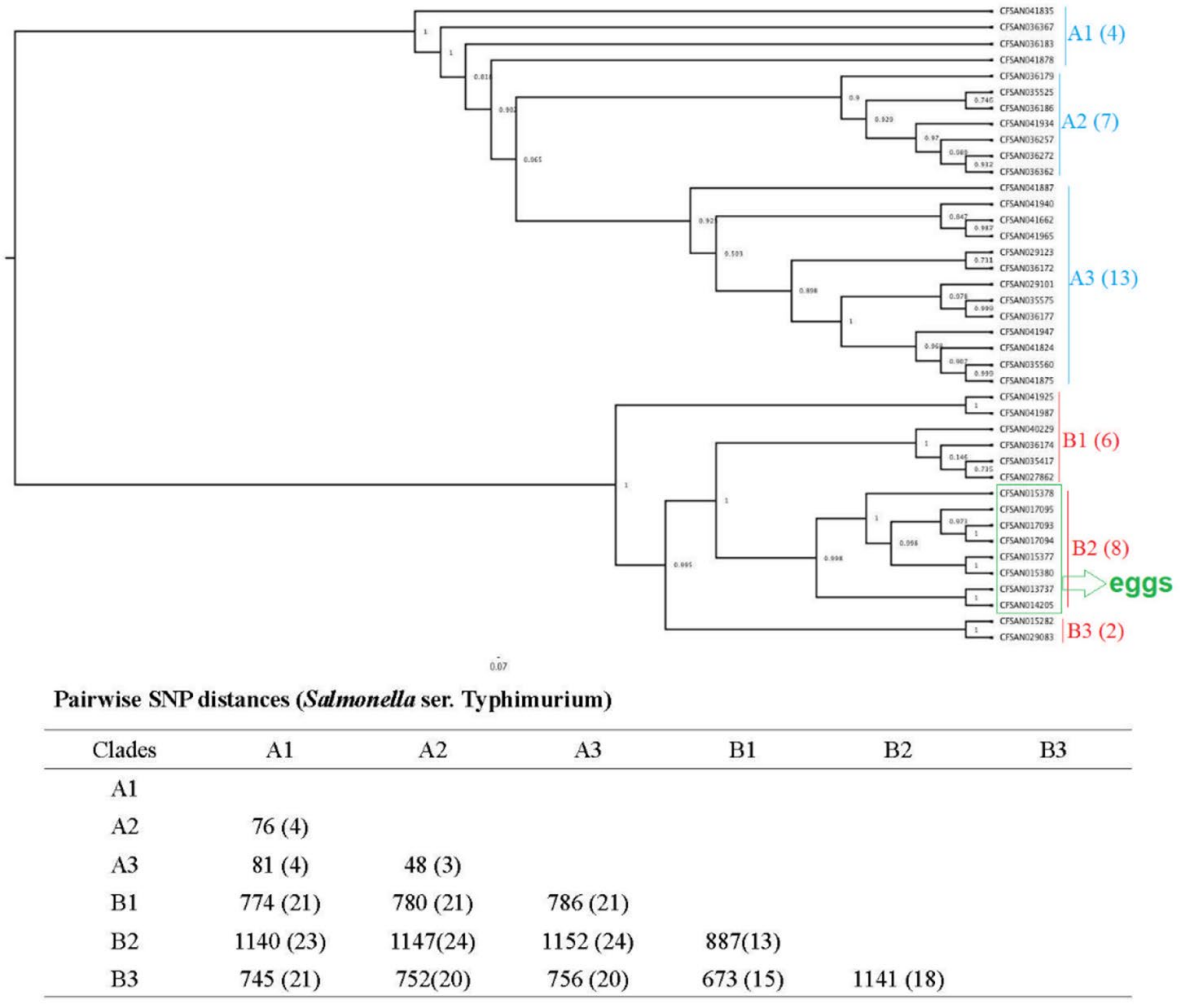
Phylogenetic tree of 40 *Salmonella* ser. Typhimurium isolates. Using core SNPs determined by FDA CFSAN SNP pipeline, the tree was constructed under the GTR-CAI model of FASTree 2. The analysis involved 673–1141 total SNP positions. Clades and subclades are indicated in colors (**A**, blue; and **B**, red) with numbers of total isolates in each clade or subclade in parentheses. Isolates sourced from eggs and clustered in subclade B2 are highlighted in green, the rest were from chicken samples.

**Figure 3:**
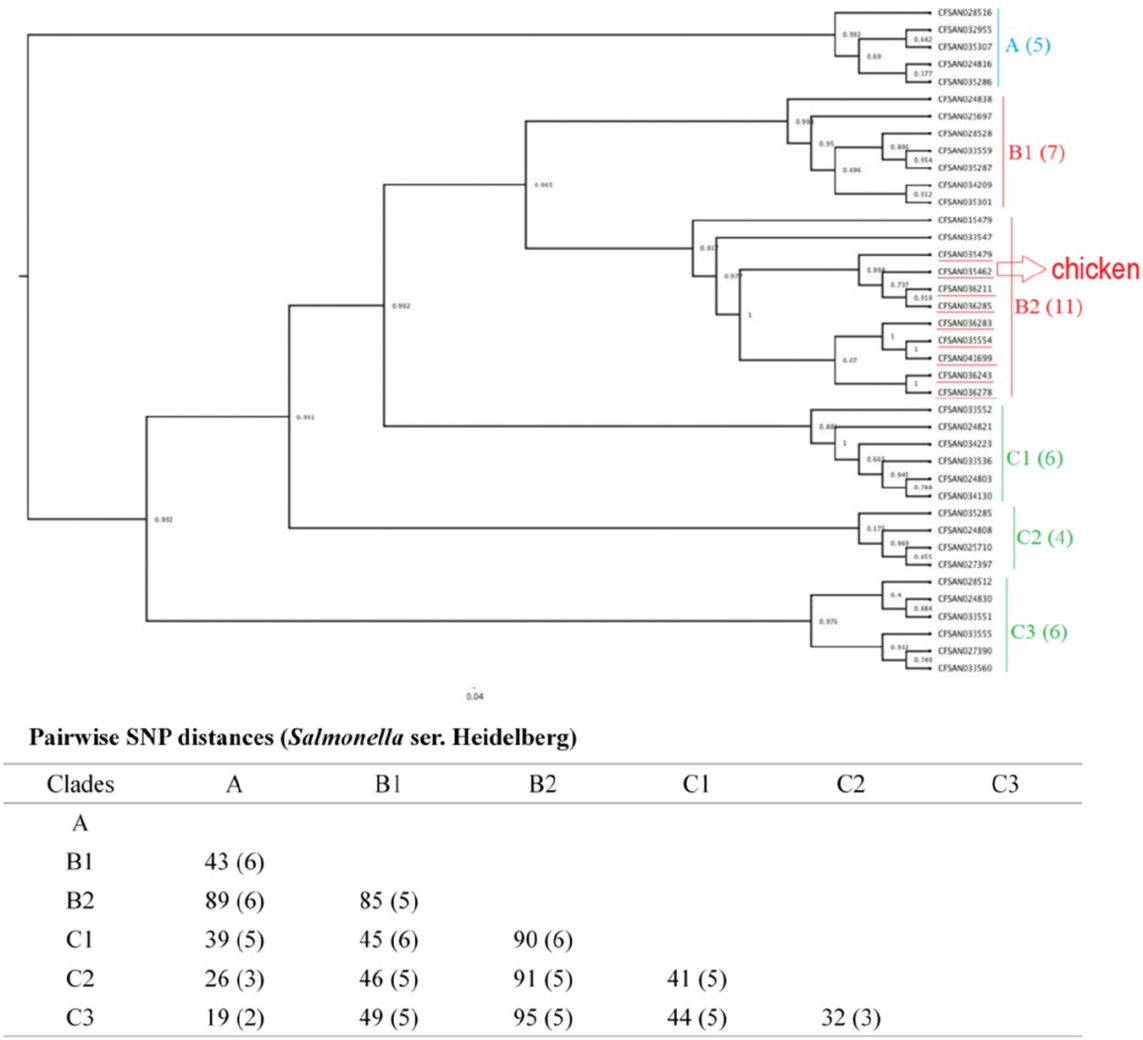
Phylogenetic tree of 39 *Salmonella* ser. Heidelberg isolates. Using core SNPs determined by FDA CFSAN SNP pipeline, the tree was constructed under the GTR-CAI model of FASTree 2. The analysis involved 19–95 total SNP positions. Clades and subclades are indicated in colors (**A**, blue; **B**, red; and **C**, green) with numbers of total isolates in each clade or subclade in parentheses. Isolates sourced from chicken and clustered in subclade B2 are highlighted in red, the rest were from egg samples.

Next, we constructed phylogenetic trees grouped by serotype to investigate the isolates’ genetic diversity. The phylogenetic tree constructed for the 64 *Salmonella* ser. Enteritidis isolates was separated into two clades (clade A and clade B) with 552–565 SNPs (Fig. 1). Forty-one of the isolates were placed into clade B with 3 subclades, namely, clade B1 (9 isolates), clade B2 (14 isolates), and clade B3 (18 isolates), with 160–219 SNPs. The 40 *Salmonella* ser. Typhimurium isolates were grouped into two clades (clade A and clade B); each clade had three subclades (clade A1, A2, A3, and clade B1, B2, B3) (Fig. 2). Within the three subclades of clade A, we identified 48–81 SNPs; within the three subclades of clade B, we identified 673–1141 SNPs. All eight *Salmonella* ser. Typhimurium isolates from egg sources were placed into clade B2. The 39 *Salmonella* ser. Heidelberg isolates were grouped in three different clades, namely, clade A, clade B, and clade C (subclade A, B1, B2, C1, C2, and C3), with less than 100 SNPs (19-95 bp) among them (Fig. 3). Five egg-sourced isolates formed clade A. Eighteen isolates belonged to clade B, and 16 belonged to clade C. Clade B2 encompassed all nine chicken-sourced and two egg-sourced (CFSAN015479 and CFSAN033547) *Salmonella* ser. Heidelberg isolates; compared to other clades, this clade also had relatively high pairwise SNP distances from other clades (85-95 bp).

### Distribution of selected target genes among three *Salmonella* serotypes

After sequencing and assembling the genomes, we performed BLAST analyses to investigate the existence of the selected target genes among *Salmonella* ser. Enteritidis, *Salmonella* ser. Typhimurium and *Salmonella* ser. Heidelberg isolates. The BLAST analyses showed that all 143 isolates contained the genes *inv*A, *ttr*RSBCA, *pho*P, *fim*A, and *agf*A (data not shown). By contrast, not all the isolates carried the *spv*C gene. Specifically, this gene was present in 59/64 *Salmonella* ser. Enteritidis isolates and in 14/40 *Salmonella* ser. Typhimurium isolates whereas none of the 39 *Salmonella* ser. Heidelberg isolates carried *spv*C. We also identified isolates that carried only partial target gene sequences. For example, two *Salmonella* ser. Typhimurium isolates (CFSAN034209, CFSAN036243) carried a partial sequence of *fim*A, and six *Salmonella* ser. Heidelberg isolates carried a partial sequence of *ttr*A (CFSAN015377, CFSAN015378, CFSAN015380, CFSAN017093, CFSAN017094, CFSAN017095).

### Mutations of the target genes

We identified numerous non-synonymous mutations and synonymous mutations among the selected target genes (Table 1). The most commonly detected non-synonymous mutations were changes between T and C (15 times), A and G (13 times), and T and G (8 times); the less frequent changes were those between G and C (4 times), A and C (4 times), and A and T (once). The mutation rates for A/T and G/C were 53.33% and 46.67%, respectively.

**Table 1:** Non-synonymous mutations observed in each target gene from all studied *Salmonella* isolates.

Genes	Non-synonymous mutation	Nucleotide (position)	NT change	Protein (position)	AA change	Associated isolates
***inv*A**	*inv*A-Mut. 1	770	C → G	257	T → S	All isolates
	*inv*A-Mut. 2	771	G → C	257	T → S	All isolates
	*inv*A-Mut. 3	898	G → T	300	A → S	All isolates
	*inv*A-Mut. 4	1012	A → T	338	T → S	All isolates
	*inv*A-Mut. 5	1125	T → G	375	D → E	All isolates
	*inv*A-Mut. 6	1198	G → C	400	V → L	All isolates
	*inv*A-Mut. 7	1201	A → G	401	S → D	All isolates
	*inv*A-Mut. 8	1202	G → A	401	S → D	All isolates
	*inv*A-Mut. 9	1301	C → T	434	A → V	All isolates
	*inv*A-Mut. 10	1302	T → G	434	A → V	All *Salmonella* ser. Enteritidis and *Salmonella* ser. Typhimurium isolates
	*inv*A-Mut. 11	1325	C → T	442	T → I	All isolates
	*inv*A-Mut. 12	1327	T → C	443	Y → H	All *Salmonella* ser. Enteritidis and *Salmonella* ser. Typhimurium isolates
	*inv*A-Mut. 13	1332	T → G	444	H → Q	All isolates
***ttr*S**	*ttr*S-Mut. 1	986	G → A	329	S → N	All *Salmonella* ser. Enteritidis isolates
	*ttr*S-Mut. 2	987	C → T	329	S → N	All *Salmonella* ser. Enteritidis isolates
	*ttr*S-Mut. 3	1117	T → G	373	C → G	All *Salmonella* ser. Enteritidis isolates
	*ttr*S-Mut. 4	1450	G → T	484	A → S	*Salmonella* ser. Enteritidis (CFSAN034231, CFSAN034232); *Salmonella* ser. Typhimurium (CFSAN017095, CFSAN017094, CFSAN017093, CFSAN015380, CFSAN015378, CFSAN015377)
	*ttr*S-Mut. 5	1465	A → G	489	I → V	All *Salmonella* ser. Enteritidis and *Salmonella* ser. Heidelberg isolates
	*ttr*S-Mut. 6	1486	A → C	496	N → H	All *Salmonella* ser. Enteritidis and *Salmonella* ser. Heidelberg isolates
	*ttr*S-Mut. 7	1502	T → C	501	V → A	All *Salmonella* ser. Enteritidis and *Salmonella* ser. Heidelberg isolates
***ttr*B**	*ttr*B-Mut.1	389	T → C	130	I → T	*Salmonella* ser. Enteritidis (CFSAN057880)
	*ttr*B-Mut.2	650	C → A	217	P → Q	*Salmonella* ser. Enteritidis (CFSAN057841, CFSAN030823, CFSAN002042)
***ttr*C**	*ttr*C-Mut.1	560	G → A	187	R → H	12 *Salmonella* ser. Typhimurium isolates^*a*^
	*ttr*C-Mut.2	740	G → T	247	C → F	All *Salmonella* ser. Enteritidis and *Salmonella* ser. Heidelberg isolates
***ttr*A**	*ttr*A-Mut.1	488	T → C	163	V → A	All *Salmonella* ser. Enteritidis isolates
	*ttr*A-Mut.2	634	C → T	212	R → C	41 *Salmonella* ser. Enteritidis isolates^*b*^
	*ttr*A-Mut.3	1024	C → G	342	Q → E	All *Salmonella* ser. Enteritidis isolates
	*ttr*A-Mut.4	2057	A → G	686	H → R	All *Salmonella* ser. Enteritidis isolates
	*ttr*A-Mut.5	2066	C → T	689	A → V	All *Salmonella* ser. Enteritidis isolates
	*ttr*A-Mut.6	2123	A → G	708	H → R	All *Salmonella* ser. Heidelberg isolates
	*ttr*A-Mut.7	2170	A → G	724	T → A	All *Salmonella* ser. Heidelberg isolates
***fim*A**	*fim*A-Mut.1	19	T → C	7	S → P	All *Salmonella* ser. Heidelberg isolates
	*fim*A-Mut.2	86	C → A	29	P → H	24 *Salmonella* ser. Typhimurium isolates^*c*^
	*fim*A-Mut.3	127	A → G	43	I → V	All *Salmonella* ser. Heidelberg isolates
	*fim*A-Mut.4	257	A → G	86	D → G	*Salmonella* ser. Enteritidis (CFSAN030097, CFSAN025700, CFSAN032858, CFSAN032959)
	*fim*A-Mut.5	319	C → T	107	P → S	*Salmonella* ser. Typhimurium (CFSAN017095, CFSAN017094, CFSAN017093, CFSAN015380, CFSAN015378, CFSAN015377)
	*fim*A-Mut.6	496	C → T	166	Q → Stop Codon	All *Salmonella* ser. Enteritidis and *Salmonella* ser. Heidelberg isolates
	*fim*A-Mut.7	630	A → C	210	Q → H	All *Salmonella* ser. Enteritidis and *Salmonella* ser. Heidelberg isolates
	*fim*A-Mut.8	709	T → C	237	Y → H	All *Salmonella* ser. Enteritidis isolates
	*fim*A-Mut.9	745	T → C	249	Y → H	All *Salmonella* ser. Heidelberg isolates
	*fim*A-Mut.10	751	T → C	251	F → L	All *Salmonella* ser. Heidelberg isolates
	*fim*A-Mut.11	794	T → G	265	V → G	All *Salmonella* ser. Enteritidis and *Salmonella* ser. Heidelberg isolates
	*fim*A-Mut.12	851	G → A	284	R → H	All *Salmonella* ser. Enteritidis isolates
	*fim*A-Mut.13	857	A → G	286	H → R	All *Salmonella* ser. Heidelberg isolates
***pho*P**	*pho*P-Mut.1	268	G → A	90	E → K	25 *Salmonella* ser. Enteritidis isolates^*d*^

^a^12 *Salmonella* ser. Typhimurium isolates: CFSAN040229, CFSAN036174, CFSAN035417, CFSAN027862, CFSAN017095, CFSAN017094, CFSAN017093, CFSAN015380, CFSAN015378, CFSAN015377, CFSAN014205, CFSAN013737.

^b^41 *Salmonella* ser. Enteritidis isolates: CFSAN057841, CFSAN035309, CFSAN035308,CFSAN035291, CFSAN035289, CFSAN035276, CFSAN035272, CFSAN034232, CFSAN034231, CFSAN034151, CFSAN033543, CFSAN033541, CFSAN032971, CFSAN032970, CFSAN032964, CFSAN032962, CFSAN032959, CFSAN032958, CFSAN030852, CFSAN030839, CFSAN030835, CFSAN030823, CFSAN030816, CFSAN030496, CFSAN030097, CFSAN030086, CFSAN030081, CFSAN030067, CFSAN030066, CFSAN028530, CFSAN027394, CFSAN027378, CFSAN027377, CFSAN025717, CFSAN025708, CFSAN025700, CFSAN024840, CFSAN024814, CFSAN017081, CFSAN002042, CFSAN058030.

^c^24 *Salmonella* ser. Typhimurium isolates: CFSAN041965, CFSAN041947, CFSAN041940, CFSAN041934, CFSAN041887, CFSAN041878, CFSAN041875, CFSAN041835, CFSAN041824, CFSAN041662, CFSAN036367, CFSAN036362, CFSAN036272, CFSAN036257, CFSAN036186, CFSAN036183, CFSAN036179, CFSAN036177, CFSAN036172, CFSAN035575, CFSAN035560, CFSAN035525, CFSAN029123, CFSAN029101.

^d^25 *Salmonella* ser. Enteritidis isolates: CFSAN035309, CFSAN035308, CFSAN035291, CFSAN035289, CFSAN035276, CFSAN035272, CFSAN033543, CFSAN033541, CFSAN032971, CFSAN032970, CFSAN032964, CFSAN032962, CFSAN030816, CFSAN030496, CFSAN030086, CFSAN030081, CFSAN030067, CFSAN030066, CFSAN028530, CFSAN027394, CFSAN027378, CFSAN025717, CFSAN025708, CFSAN024840, CFSAN024814.

Regarding the mutations in each gene, we identified 13 unique non-synonymous mutations and 80 synonymous mutations in *inv*A across all our isolates (data not shown). Although the mutations *inv*A-Mut.1 and *inv*A-Mut.2 were changes of C → G (nt position 770) and G → C (nt position 771), respectively, they resulted in the same amino acid (AA) change in *inv*A protein, from threonine (T) to serine (S), at site 257 (Table 1).

In the *ttr*S, *ttr*B, *ttr*C, and *ttr*A genes we found 7, 2, 2, and 7 non-synonymous mutations, respectively. Most non-synonymous mutations in *ttr*SBCA occurred in the *Salmonella* ser. Enteritidis and *Salmonella* ser. Heidelberg isolates. We did not identify non-synonymous mutations in the *ttr*B and *ttr*A genes among the *Salmonella* ser. Typhimurium isolates. Six *Salmonella* ser. Typhimurium isolates had a unique non-synonymous mutation in the *ttr*S gene (*ttr*S-Mut.4), and 12 *Salmonella* ser. Typhimurium isolates had a non-synonymous mutation in the *ttr*C gene (*ttr*C-Mut.1). We identified four *Salmonella* ser. Enteritidis isolates that carried mutations in *ttr*B (*ttr*B-Mut.1: CFSAN057880; *ttr*B-Mut.2: CFSAN057841, CFSAN030823, CFSAN002042). Both *ttrS*-Mut.1 (nt position 986, G → A) and *ttrS*-Mut.2 (nt position 987, T → C) resulted in an AA change of serine (S) → asparagine (N). No mutations were found in *ttr*R among all the isolates studied.

The *fim*A gene was found to have 13 non-synonymous mutations although most of them were identified in the *Salmonella* ser. Enteritidis and *Salmonella* ser. Heidelberg isolates; only two synonymous mutations separately found in one *Salmonella* ser. Enteritidis isolate (CFSAN030097, raw egg yolks) and in all *Salmonella* ser. Heidelberg isolates (data not shown). Notably, all *Salmonella* ser. Enteritidis and *Salmonella* ser. Heidelberg isolates carried one common nonsense mutation (*fim*A-Mut.6), C → T (nt position 496), resulting in a change at AA 166, namely, glutamine (Q) → stop codon (TAG).

Finally, the *pho*P gene exhibited one non-synonymous mutation across 25 *Salmonella* ser. Enteritidis isolates and four synonymous mutations among all *Salmonella* ser. Enteritidis isolates; no *pho*P mutation was found among the isolates in the other two serotypes. For the *agf*A and *spv*C genes, only six synonymous mutations occurred in the *Salmonella* ser. Heidelberg and *Salmonella* ser. Typhimurium isolates, and one synonymous mutation of the *spv*C gene occurred in *Salmonella* ser. Enteritidis isolates (data not shown). No non-synonymous mutations were observed for the *agf*A and *spv*C genes among the isolates studied.

### Phylogenetic analyses for each target gene

**Figure 4:**
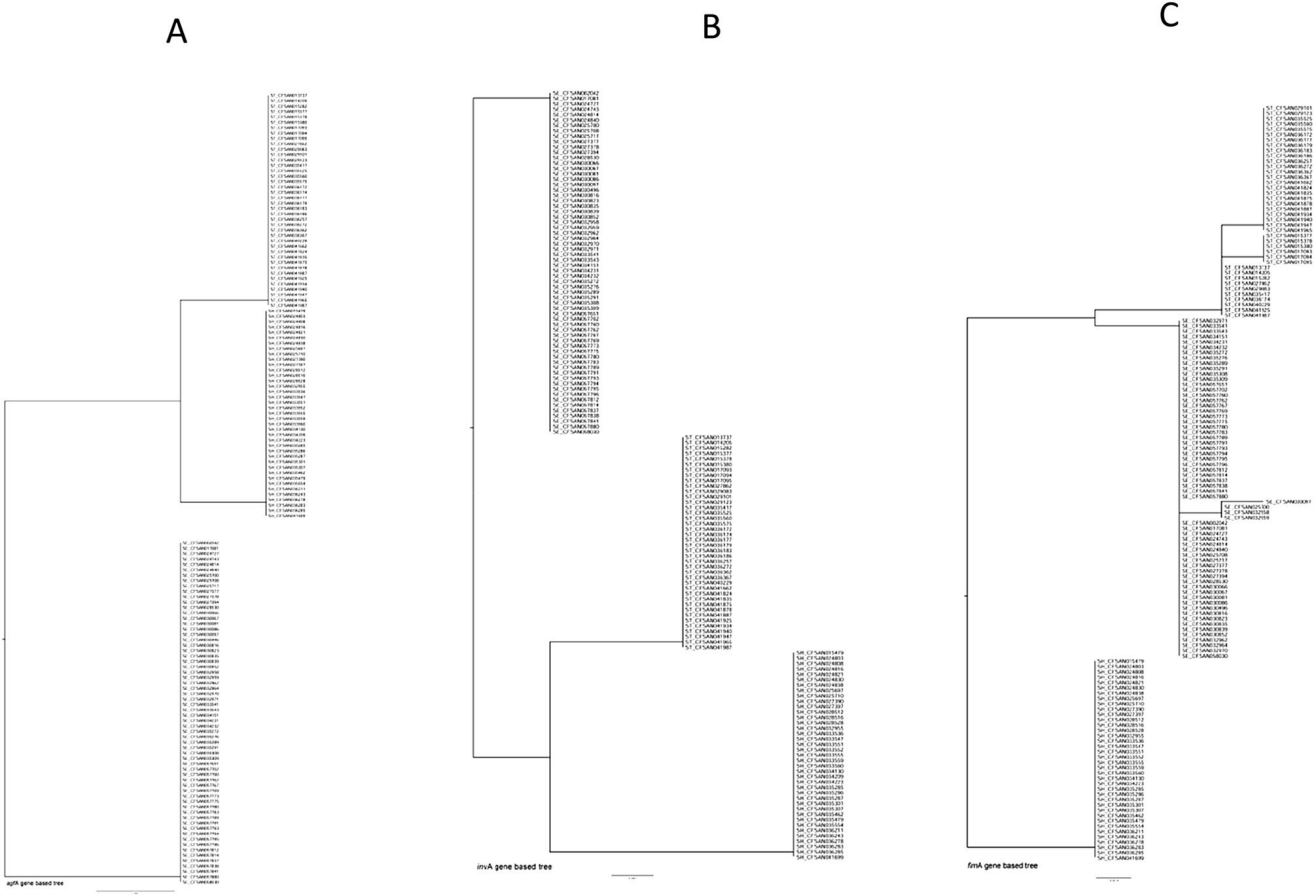
Phylogenetic trees of all sequenced *Salmonella* isolates based on nucleotide sequences of the target genes *agf*A (**A**), *inv*A (**B**), and *fim*A (**C**). The trees were constructed by the neighbor jointing method using CLC Genomics Workbench. The scale indicates the sequence percentage of base distance (percent divergence).

**Figure 5:**
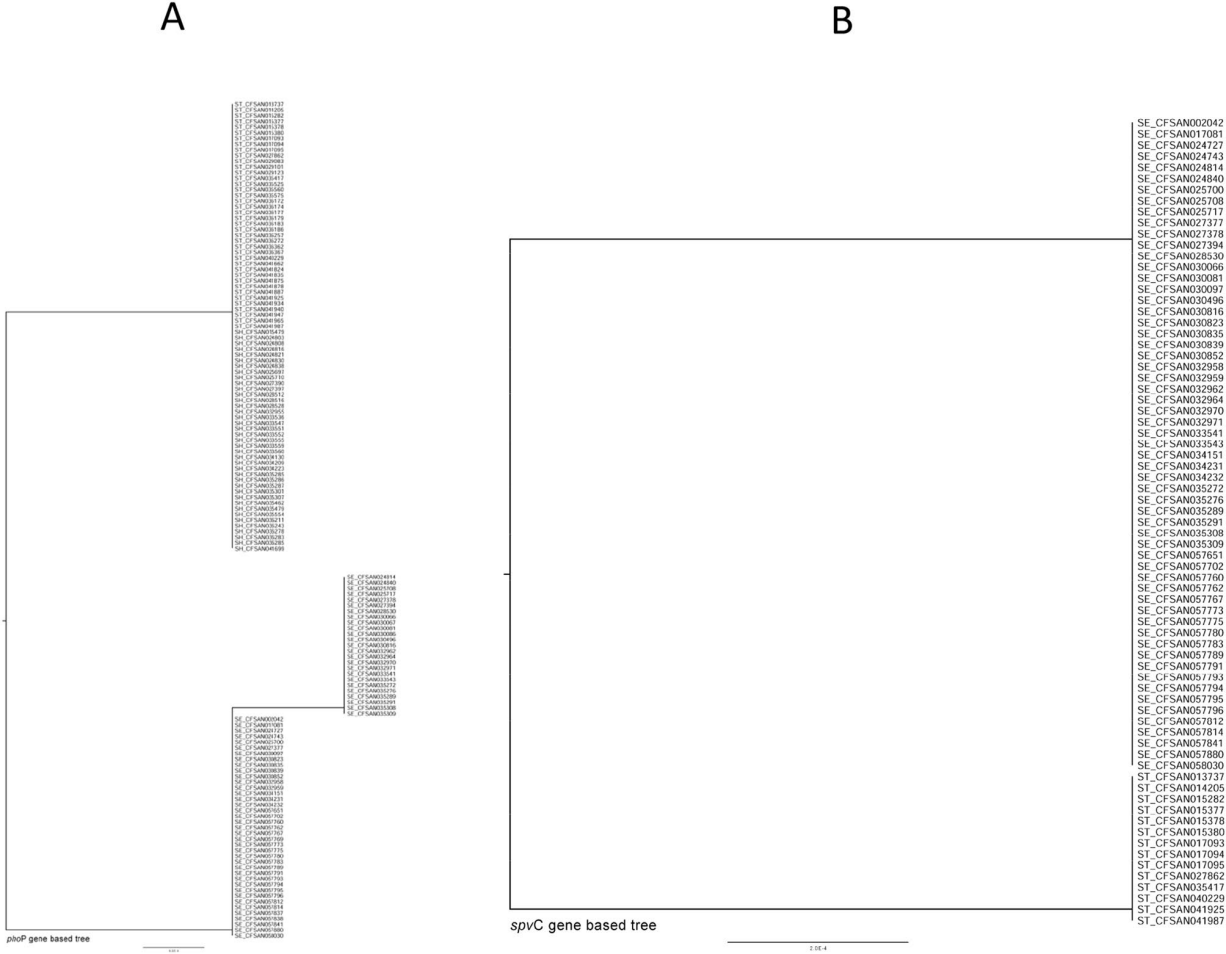
Phylogenetic trees of all sequenced *Salmonella* isolates based on the nucleotide sequences of the target genes *pho*P (**A**) and *spv*C (**B**). The trees were constructed by the neighbor jointing method using CLC Genomics Workbench. The scale indicates the sequence percentage of base distance (percent divergence).

**Figure 6:**
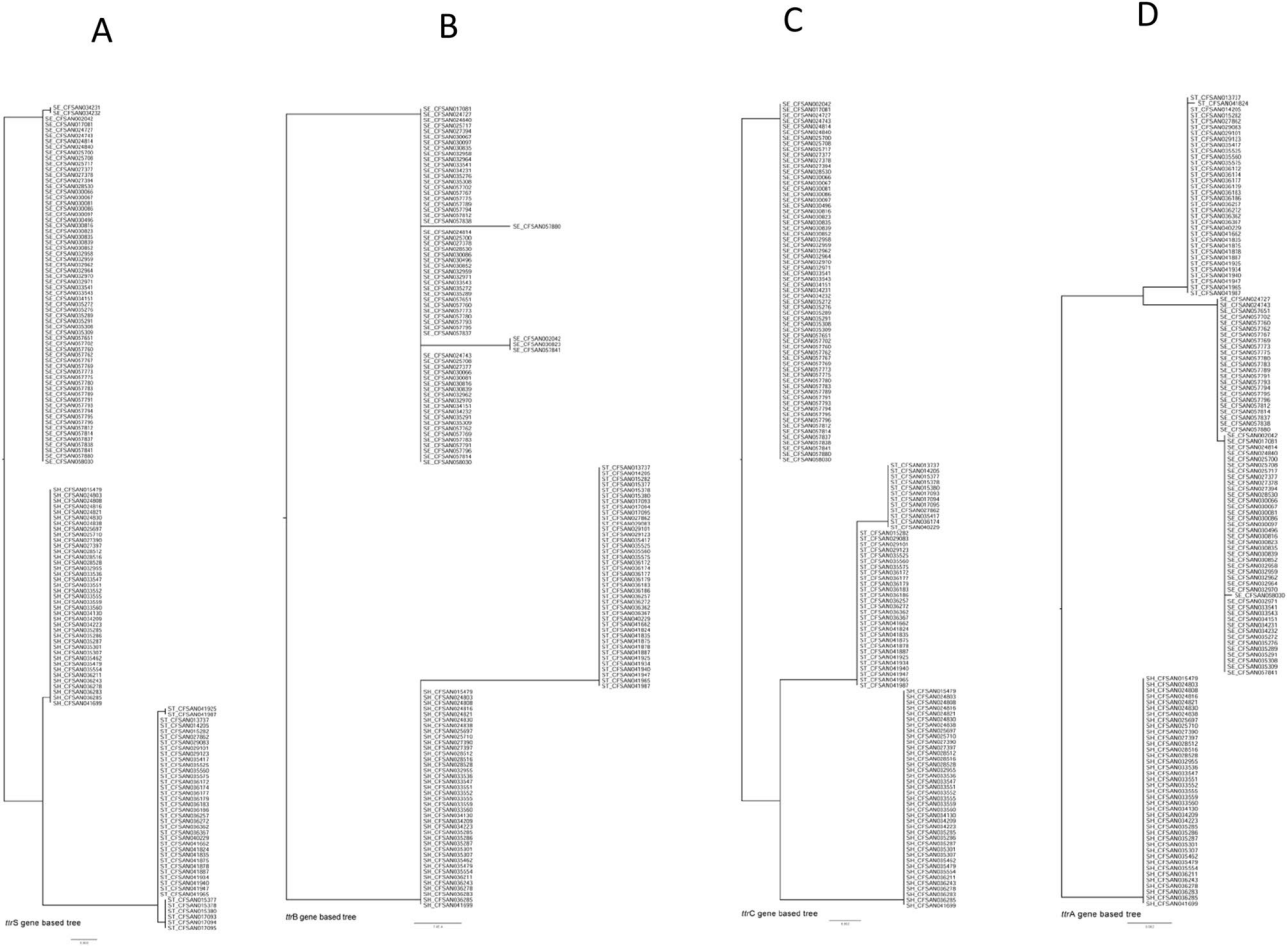
Phylogenetic trees of all sequenced *Salmonella* isolates based on the nucleotide sequences of the target genes *ttr*S (**A**), *ttrB* (**B**), *ttrC* (**C**), and *ttrA* (**D**). The trees were constructed by the neighbor jointing method using CLC Genomics Workbench. The scale indicates the sequence percentage of base distance (percent divergence).

Phylogenetic trees were then constructed for each of the selected target genes based on their nucleotide sequences (including synonymous/non-synonymous mutations) in the *Salmonella* isolates studied to investigate their genetic differences. We found disparities among all selected target genes (Figs. 4, 5, 6) across all three serotypes except *ttrR* gene. Four *Salmonella* ser. Enteritidis isolates (CFSAN030097, CFSAN025700, CFSAN032958, CFSAN032959) were different from the others based on the phylogenetic tree of the *fim*A gene, and the *Salmonella* ser. Typhimurium isolates were separated into three clusters (Fig. 4C). In the *pho*P-based phylogenetic tree, the isolates of *Salmonella* ser. Typhimurium and *Salmonella* ser. Heidelberg were in the same clade, and *Salmonella* ser. Enteritidis isolates were divided into two different clades (Fig. 5A). Variations were observed for the phylogenetic trees constructed for *ttr*S (Fig. 6A), *ttr*B (Fig. 6B), *ttr*C (Fig. 6C) and *ttr*A (Fig. 6D). As our set of isolates did not exhibit mutations in *ttr*R, we could not generate a tree based on that target gene.

### Protein structure prediction

**Figure 7:**
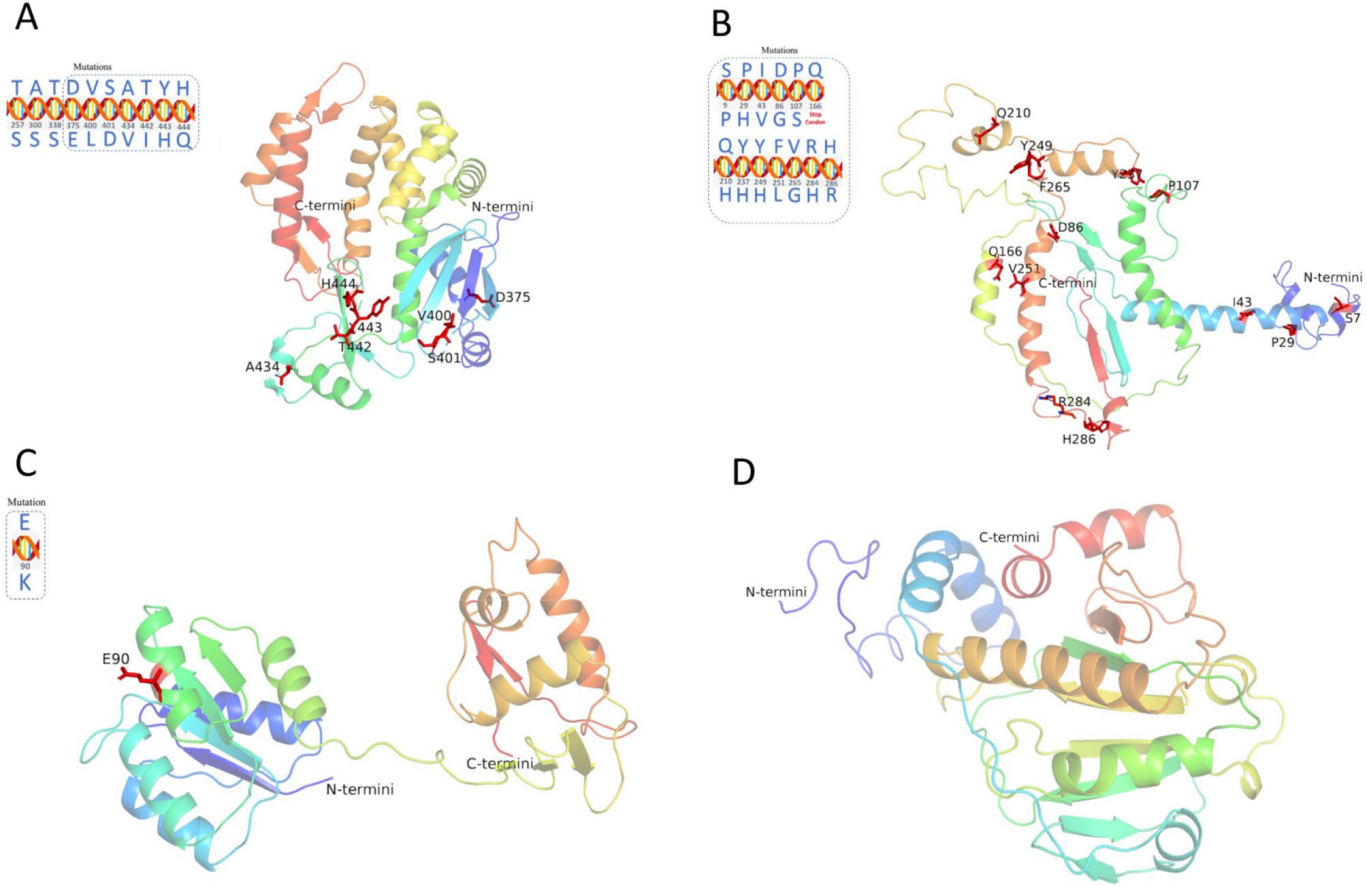
Predicted protein structures of the target genes *inv*A (**A**), *fim*A (**B**), *pho*P (**C**), and *spv*C (**D**). Positions of amino acid mutations in each target protein are shown in the circled box and labeled in red in the predicted model.

**Figure 8:**
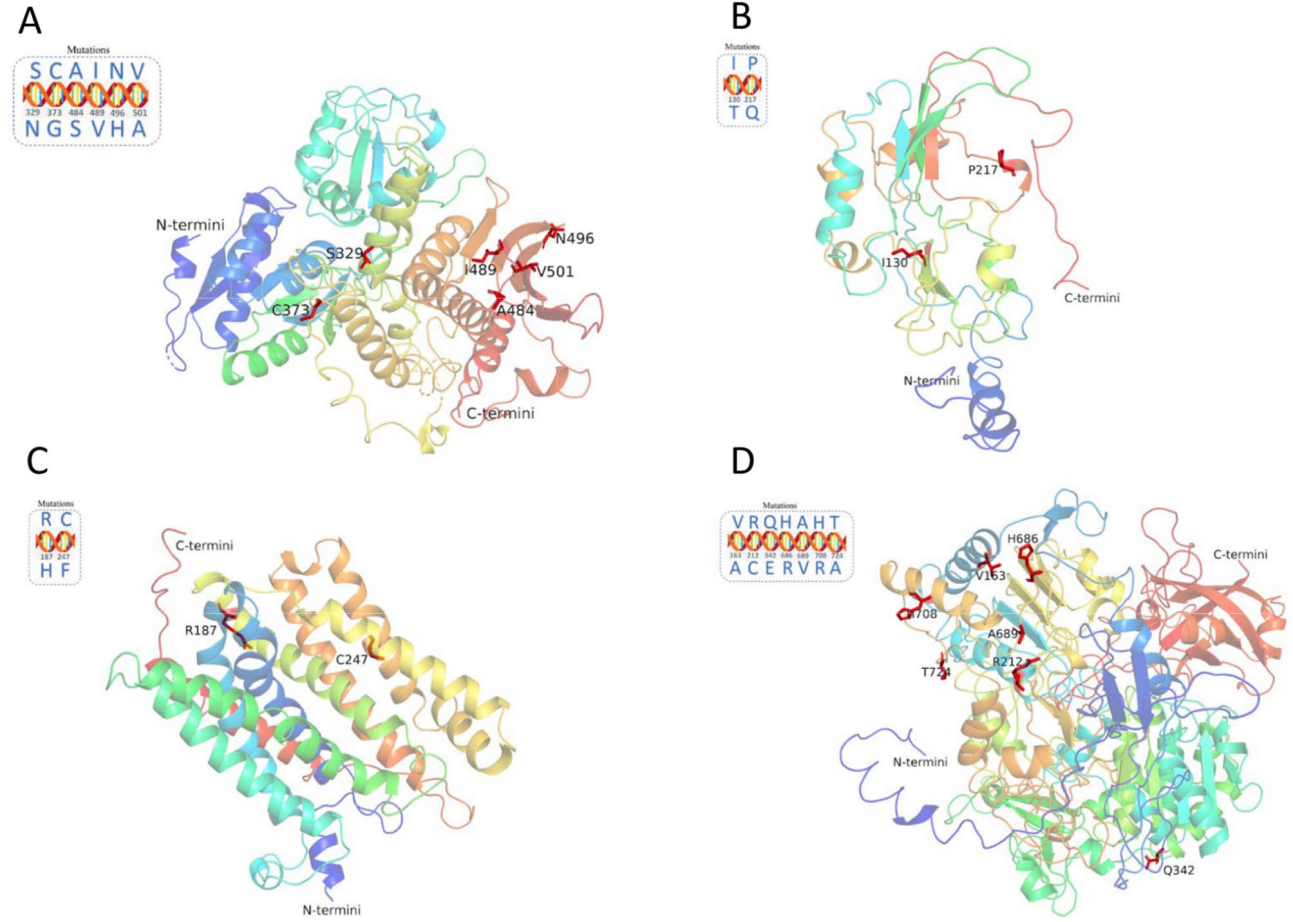
Predicted protein structures of the target genes *ttr*S (**A**), *ttrB* (**B**), *ttrC* (**C**), and *ttrA* (**D**). Positions of amino acid mutations in each target protein are shown in the circled box and labeled in red in the predicted model.

The protein structures for the genes *pho*P (Fig. 7C), *spv*C (Fig. 7D), *ttr*S (Fig. 8A), *ttr*B (Fig. 8B), *ttr*C (Fig. 8C), and *ttr*A (Fig. 8D) were successfully predicted with high confidence (> 90%). The predicted protein structure of the *inv*A gene showed a good Global Model Quality Estimation (GMQE) score of 0.43 and Qualitative Model Energy Analysis (QMEAN) score of 1.01, which covered 7 of the AA mutations revealed (Fig. 7A). Although, we tried to predict the protein structure resulting from the *fim*A gene using Phyre^2^ and SWISS-MODEL, both software programs failed to generate an acceptable result. The predicted protein structure for the *fim*A gene, as generated by SWISS-MODEL, had a GMQE score of 0.99, but the low QMEAN score (-5.44) suggested that it may not be the best representation possible (Fig. 7B). However, the structure generated by Phyre^2^ was worse and exhibited only less than 21% confidence (model not shown). Similarly, we did not finalize models for the protein structures of the *agf*A and *ttr*R genes, as the confidence and quality scores from both Phyre^2^ and SWISS-MODEL were too low to proceed. Additionally, from the predicted structure (Figs. 7 and 8), evidently the two common secondary structures α-helix and β-sheet existed in each gene: *inv*A (8 α-helixes and 5 β-sheets), *fim*A (8 α-helixes and 2 β-sheets), *pho*P (8 α-helixes and 4 β-sheets), *spv*C (6 α-helixes and 3 β-sheets), *ttr*S (14 α-helixes and 6 β-sheets), *ttr*B (4 α-helixes and 3 β-sheets), *ttr*C (12 α-helixes and 0 β-sheets), and *ttr*A (15 α-helixes and 12 β-sheets).

## Discussion

### Phylogenetic analyses of three *Salmonella* serotypes

WGS is the most powerful tool for bacterial genomic variation analyses because it is based on the complete bacterial genome at DNA level. WGS SNP analysis was used to ascertain that a new lineage of *Salmonella* ser. Enteritidis occurred and spread in Brazil after 1994 by investigating 256 *Salmonella* ser. Enteritidis isolates obtained over 48 years^23^. In Denmark, reanalysis of isolates in eight previously reported outbreaks by WGS successfully discriminated 372 isolates of *Salmonella* ser. Typhimurium and its monophasic variants^24^. Allard et al. (2013) sequenced and analysed 106 *Salmonella* ser. Enteritidis isolates from clinical, food and farm environments related to the 2010 shell egg outbreak in the U.S.; these isolates were classified into nine lineages and had a range of 100 to 600 diverse SNPs^25^. Across the 143 genomes of *Salmonella* Enteritidis, Typhimurium and Heidelberg analyzed in the current report, *Salmonella* ser. Typhimurium displayed the highest degree of genetic diversity, with a distance of 48–1152 SNPs among the different clades, while the value for *Salmonella* ser. Enteritidis was 160–565 SNPs, and that for *Salmonella* ser*.* Heidelberg was 19–95 SNPs (Figs. 1, 2, 3). Our results agree with a previous study in which *Salmonella* ser. Typhimurium had the highest phylogenetic diversity compared with *Salmonella* ser. Newport and *Salmonella* ser. Dublin^26^. *Salmonella* ser. Typhimurium and its monophasic variant have quite distinctive evolutive pathways (exploration–exploitation pathways) in comparison with *Salmonella* ser. Enteritidis and *Salmonella* ser*.* Heidelberg, which could be one of the explanations for this phenomenon^27^. Interestingly, in this study, all eight *Salmonella* ser. Typhimurium isolates from egg sources were placed into the same subclade (clade B2), whereas five egg sourced *Salmonella* ser. Heidelberg grouped into one clade (clade A) and nine chicken sourced isolates and two egg sourced isolates placed into the same subclade (clade B2). Unfortunately, there was limited epidemiological information available for these isolates to explore the relationships among these isolates.

### DNA sequence variation of target genes among *Salmonella* isolates and their phylogenetic relationships

The selected target genes/gene clusters *inv*A, *ttr*RSBCA, *pho*P, *fim*A, *agf*A, and *spv*C are all virulence factors of *Salmonella*. The *spv*C gene is on the *Salmonella* virulence plasmid, while the others are located on chromosomes^28^*.* Although the *spv*ABCD gene cluster was reported to be highly conserved in *Salmonella* ser. Typhimurium, *Salmonella* ser. Dublin and *Salmonella* ser. Choleraesuis^29^, our analysis revealed that the *spv*C gene was primarily found among *Salmonella* ser. Enteritidis isolates (92.19%), followed by *Salmonella* ser. Typhimurium isolates (35%). None of our *Salmonella* ser. Heidelberg isolates carried *spv*C, which is consistent with previous reports^30–33^. Study had shown that *spv*C is essential for the virulence of *Salmonella* ser. Typhimurium in mice; the potential impact of the presence and absence of the *spv*C gene on the isolates’ ability of causing infection warrants further investigation^55^. The *ttr*R gene was the most highly conserved compared to the other *ttr* genes, as no mutations were found in *ttr*R among all the isolates studied. In addition, only selected alleles which were more frequently used in detection technology design of the selected target genes/gene clusters were investigated in the current project.

Analysis of the phylogenetic diversity of the serotypes based on the phylogenetic trees constructed using individual genes, showed that the serotypes differ in the genes *ttr*S, *ttr*B, *ttr*C, and *ttr*A (Fig. 6). Variations were observed in *Salmonella* ser. Enteritidis and *Salmonella* ser. Typhimurium, but the genes in *Salmonella* ser. Heidelberg were more stable. The *ttr*R gene was the most highly conserved compared to the other *ttr* genes, because no mutations were found in *ttr*R among all the isolates studied. As a gene cluster within the *Salmonella* pathogenicity island (SPI, part of the flexible gene pool) on the chromosome, the *ttr*RSBCA locus has been shown to be transferable through horizontal gene transfer (HGT) events such as transfer by phages or conjugative transposons. This might be one of the reasons that many PCR protocols have an extra target (such as *inv*A) in combination with the *ttr*RSBCA gene to reinforce the specificity of PCR detection^9,34^. In addition, the presence of mutations in these genes could also be an underlying reason.

Finally, our study also demonstrated the presence of partial *fim*A (two isolates) and *ttr*A (six isolates) gene sequences among some *Salmonella* isolates and all studied *Salmonella* isolates contained the genes *inv*A, *pho*P, and *agf*A. Phylogenetic trees generated based on both the *inv*A and *agf*A genes clearly comprised three lineages of three serotypes (Fig. 4A and B). Interestingly, the *pho*P-based phylogenetic tree divided the *Salmonella* ser. Enteritidis isolates into two different clades (Fig. 5A). In this case, along with the analysis of the *fim*A, *spv*C, and *ttr*RSBCA genes (Figs. 4C, 5B, 6), we revealed that the *inv*A, *pho*P, and *agf*A genes have higher discriminatory power to diagnose *Salmonella* at the genus level than other selected genes.

### Mutations of target genes and prediction of protein structures

Among the 143 *Salmonella* isolates investigated in this study, both synonymous and non-synonymous mutations in the selected target genes were discovered. Among the total 45 non-synonymous mutations observed (Table 1), the top two mutations occurred with an AA change between the T and C alleles (15 times) and the A and G alleles (13 times). The substitution rates of A/T and G/C were approximately equal. Similarly, in a study involving 106 *Salmonella* ser. Enteritidis isolates, 55 non-synonymous mutations were discovered, and the top two mutations also occurred between the alleles T ↔ C (27 times) and A ↔ G (15 times)^25^. These results are in accordance with the biased gene conversion model, in which AT → GC mutations have a higher probability of being transmitted to the next generation, as an AT/GC heterozygote produces more gametes carrying G or C than those carrying A or T, presumably through the GC-biased repair of A:C and G:T mismatches in heteroduplexed recombination intermediates^35,36^. In addition, polymorphisms in an organism result from mutation, selection, and other processes such as biased gene conversion, which favors the transmission of G/C over A/T alleles^35,36^.

In the successfully predicted protein structures of the target genes (Figs. 7 and 8), the overwhelming majority of the mutations were located in the main domain of the structure, except for *fim*A-Mut.1, which occurred close to the C-terminal, and *fim*A-Mut.13, which occurred close to the N-terminal. The mutations mainly occurred in the α-helix and β-sheet which are key elements for maintaining the conformation of proteins, therefore, such mutations could interfere with the hydrogen bonding between main-chain amide and carbonyl groups and their corresponding representations. It is well known that C-terminal sequence is an important structural and functional site of proteins and peptides whereas N-terminal influences the overall biological function of the protein.

The *inv*A gene encodes an N-terminal integral membrane domain and a C-terminal cytoplasmic domain that is proposed to form part of a docking platform. As *inv*A is essential for *Salmonella* to gain access to epithelial cells, isolates with non-synonymous *inv*A mutant may have reduced virulence^37,38^. Since *inv*A gene has been well acknowledged as an effective target to detect *Salmonella,* it seems that the gene mutations happened to *inv*A have less impact on the protein functions. It will take further experiments and data to prove this theory and reveal the mechanisms.

In terms of the number of mutations present in *ttr*RSBCA, we speculated that the *ttr*R gene was the most highly conserved, followed by the *ttr*B and *ttr*C genes, which have been successfully used in real-time PCR detection of *Salmonella* in food^10,34^. Differences in the prevalence of mutations in the *ttr*RSBCA cluster may be related to the distinct functions and loci of these genes. The *ttr*R and *ttr*S genes are components of the *ttr*SR two‐component regulatory system for functional tetrathionate reductase expression, while *ttr*A, *ttr*B and *ttr*C are tetrathionate reductase structural genes. The *ttr*B iron–sulphur clusters probably function in the transfer of electrons from *ttr*C to *ttr*A^39^.

Among the 64 *Salmonella* ser. Enteritidis isolates studied, 25 had one mutation each in the *pho*P gene (G → A, nt 268). Allard et al. (2013) also observed the same non-synonymous mutation in the *pho*P gene in *Salmonella* ser. Enteritidis isolates from egg-associated samples^25^. The *pho*P protein has a conserved N-terminal domain with an essential aspartate residue and a C-terminal domain that binds DNA. The *pho*P/Q two-component system, encoded by *pho*P and *pho*Q, controls more than 40 genes, such as *prg*s, *pag*O, *pag*C, and *pag*D, which regulate the host inflammatory response, lipopolysaccharide (LPS) formation, and extracellular protein transport and promote virulence and intracellular survival^40–43^. This system may also play a specific role in *Salmonella* ser. Enteritidis pathogenicity in mice^44^. A *pho*P-based LAMP assay has been developed to effectively detect *Salmonella* in food samples^13^. The stable peculiarity (one mutation has been found) of *pho*P gene also demonstrated the potential of *pho*P gene as target gene for detecting *Salmonella*.

It was reported that the *fim*A gene contains sequences unique to *Salmonella* strains and is an effective target for detecting *Salmonella* in feed and food samples^16,45^. However, in this study, we not only found the *fim*A mutations happened close to N/C-terminal, but all *Salmonella* ser. Enteritidis and *Salmonella* ser. Heidelberg isolates carried one common nonsense mutation (*fim*A-Mut.6, Q → stop codon), which indicated that the *fim*A gene has questionable sites for being used as a target of method design to detect *Salmonella*. The *fim*A gene, encoding a major fimbria unit, was mapped within the *fim* gene cluster for the chaperone–usher pathway for the assembly and secretion of multi-subunit appendages (type I pili/fimbriae). The type I pili consists of a helical rod-like structure (*fim*A and *pap*A) and a flexible tip that contains the minor pilus subunits (*fim*F, *fim*G and *fim*H, *pap*E, *pap*F, *pap*G, *pap*K). Solved crystal structures have shown the elongation complex *fim*D–*fim*H–*fim*G–*fim*F–*fim*C and the next subunit–chaperone complex of *fim*A–*fim*C in the chaperone–usher pathway. The 3D structure of *fim*A modelled on a *fim*H-G1 template indicates that the interface between the subunits contains small hydrophobic or polar residues such as alanine (A), serine (S) and threonine (T)^46–48^. This may provide an explanation for the revealed non-synonymous mutation at the N-terminus (S → P) of the predicted *fim*A structure.

No non-synonymous mutations were detected in the *agf*A and *spv*C genes. Our attempt to predict protein structure of *agf*A was unsuccessful. Limited reports on *agf*A gene call for further research on this gene. The *agf*BCA operon encodes thin aggregative fimbriae/curli (formerly SEF17), and the thin aggregative fimbriae are primarily comprised of *agf*A subunits^49,50^. Although thin aggregative fimbriae are produced by most *Salmonella* and *Escherichia coli* isolates^51^ and a high thin aggregative fimbriae sequence similarity was found between *Salmonella* ser. Enteritidis SEF17 fimbriae and *E. coli* curli^49^. Doran et al. (1993) reported that *agf*A-based nucleotide probes hybridized only to *Salmonella* DNA^52^. The *agf*A gene has been successfully used as a target for *Salmonella* detection^9^. The *spv* genes, including *spv*A, *spv*B, *spv*C, *spv*D, and *spv*R, are often carried on a large *Salmonella* virulence plasmid. However, in some serotypes, they are integrated into the chromosome^53^. The *spv*C gene, as a *Salmonella* effector with phosphothreonine lyase activity towards host mitogen‐activated protein kinases, can be secreted in vitro by the SPI‐1 and SPI‐2 type III secretion systems^54^. This gene is essential for the full virulence of *Salmonella* ser. Typhimurium in mice^55^. Oligonucleotide insertions in *spv*C were shown to be nonpolar^56^.

Currently, limited information is known about the complex functions of the target genes frequently used for the detection of the above mentioned *Salmonella* isolates. Our study provided comparison of 10 target genes from the perspective of DNA sequence and protein structure. More efforts should be made to determine whether these mutations can further affect the protein functions. And further biological, molecular, and functionality research would help fill the knowledge gaps in this area. We found both non-synonymous and synonymous mutation rates vary among the target genes frequently used based on the three serotypes studied. Large scale investigation of more serotypes regarding mutation rates are needed to determine which genes are more suitable for use as detection targets. With the use and sharing of this new information about these target genes in the future, the ability to identify and investigate *Salmonella* infections by comparing gene sequence data will be greatly enhanced.

## Materials and methods

### *Salmonella* isolates

We selected 143 *Salmonella* isolates from chicken/duck eggs (including raw egg whites, raw egg yolks, raw whole eggs, pecked eggs, egg slurry, egg salad, frozen liquid egg, cooked quail eggs, salted duck eggs, duck egg yolks, frozen salted duck yolks) and chicken (chicken, chicken jerky, chicken breast): 64 *Salmonella* ser. Enteritidis isolates were collected from 1995 to 2016 (Supplementary Table 1) as described previously^57^; 40 *Salmonella* ser. Typhimurium isolates were collected from 2001 to 2010 (Supplementary Table 2); and 39 *Salmonella* ser. Heidelberg isolates were collected from 2003 to 2013 (Supplementary Table 3). The isolates were originally collected in the U.S., Brazil, China, and Chile. All isolates were from the historical collection of the Division of Microbiology (DM), Office of Regulatory Science (ORS), Center for Food Safety and Applied Nutrition (CFSAN), U.S. FDA. Serotypes of the isolates were reconfirmed using the Kauffmann and White classification scheme (https://www.pasteur.fr/sites/default/files/veng_0.pdf). All isolates were cultured overnight at 37 ± 2 °C in tryptic soy broth (Becton Dickinson, Franklin Lakes, NJ, USA) for DNA extraction.

### Whole genome sequencing and creation of phylogenetic trees

Genomic DNA from each isolate was extracted using the DNeasy Blood and Tissue Kit (Qiagen, Valencia, CA) and quantified using a Qubit 3.0 fluorometer (Life Technologies, MD). Libraries were prepared according to Nextera XT protocols and then sequenced on the Illumina MiSeq/NextSeq 500 platform using the NextSeq 500/550 High Output Kit v2 following the manufacturer’s instructions (Illumina, San Diego, CA). All genomes were sequenced with 300 cycles, pair-end library with coverage depth of > 30 × at FDA CFSAN. The Illumina reads were assembled de novo using CLC Genomics Workbench v9 (Qiagen Bioinformatics, Redwood City, CA). The Sequence Read Archive (SRA) Toolkit 2.8.1–3 (https://trace.ncbi.nlm.nih.gov/Traces/sra/?view=software) was used to download and convert SRR files to FASTQ files. Using the FDA CFSAN SNP pipeline^58^, we acquired the SNP matrix and generated the phylogenetic tree under the GTR + CAT model of FASTtree2^59^. The complete genomes of *Salmonella* ser. Enteritidis P125109 (NC_011294.1), *Salmonella* ser. Typhimurium LT2 (NC_003197.2), and *Salmonella* ser. Heidelberg A3ES40 (NZ_CP016561.1) were utilized as the references for constructing the phylogenetic trees of *Salmonella* ser. Enteritidis*, Salmonella* ser. Typhimurium*,* and *Salmonella* ser. Heidelberg, respectively. FigTree software was used to visualize the generated phylogenetic trees (http://tree.bio.ed.ac.uk/software/figtree/). The WGS data of all the isolates studied here can be accessed from the NCBI SRA (https://www.ncbi.nlm.nih.gov/sra). Their accession numbers are listed (Supplementary Tables 1–3).

### Identifying nucleotide mutations and predicting the protein structures of the selected genes

The DNA sequences of the genes *inv*A*, ttr*RSBCA, *pho*P, *fim*A, *agf*A and *spv*C from each isolate were aligned using MEGA v7 (https://www.megasoftware.net/) to the reference genes from NCBI: *inv*A (GenBank: DQ644631.1), *ttr*RSBCA (GenBank: AF282268.1), *pho*P (GenBank: NC_003197.2, 1,318,679—1,319,365), *fim*A (GenBank: M18283.1), *agf*A (GenBank: U43280.1), and *spv*C (GenBank: HM044662.1). Protein structure prediction for *inv*A and *fim*A was performed with SWISS-MODEL (Swiss Institute of Bioinformatics, Basel, Switzerland)^60^, and that for other genes was performed using Phyre^2^ (Structural Bioinformatics Group, Imperial College, London)^61^; all the structure illustrations were drawn using PyMOL v2.2 (https://pymol.org/2/).

## Supplementary Information

Supplementary Information.
