# Supplementary material for: Whole genome sequencing and protein structure analyses of target genes for the detection of Salmonella
Source: Sci Rep. 2021 Oct 22;11:20887. doi: 10.1038/s41598-021-00224-7 (PMC8536731; doi:10.1038/s41598-021-00224-7)
Supplement: Supplementary file 1 — Supplementary Information. [file 41598_2021_224_MOESM1_ESM.docx]

**Running title:** Target genes for *Salmonella* detection

**Whole genome sequencing and protein structure analyses of target genes for the detection of *Salmonella***

Lijun Hu^a^, Guojie Cao^a^, Eric W. Brown^a^, Marc W. Allard^a^, Li M. Ma^b^ and Guodong Zhang^a,*^

^a^ Division of Microbiology, Office of Regulatory Science, Center for Food Safety and Nutrition, U.S. Food and Drug Administration, College Park, MD

^b^ National Institute for Microbial Forensics & Food and Agricultural Biosecurity, Department of Entomology and Plant Pathology, Oklahoma State University, Stillwater, OK

**^*^ Corresponding author:** Dr. Guodong Zhang

**Phone:** 240 - 402 - 2943; **Fax:** 301 - 436 - 2644

**E-mail:** Guodong.Zhang@FDA.HHS.GOV

**Mailing address:** Center for Food Safety and Applied Nutrition, Food and Drug Administration, 5001 Campus Dr., College Park, MD 20740

**Supplementary Table 1. Sources of each *Salmonella* ser. Enteritidis isolate and their *de novo* assembled genomes.**

| **Isolates** | **Source** | **Location***^a^* | **Year** | **Genome**  **Size (Mb)** | **N50 (Mb)** | **# of contigs** | **Depth of coverage** |
| --- | --- | --- | --- | --- | --- | --- | --- |
|  |  |  |  |  |  |  |  |
| CFSAN057702 | Eggs | Brazil | 2000 | 4.70 | 0.21 | 51 | 220 |
| CFSAN057880 | Egg | Brazil | 2016 | 4.74 | 0.19 | 60 | 181 |
| CFSAN025700 | Whole eggs | USA:IN | 2012 | 4.71 | 0.49 | 30 | 143 |
| CFSAN024840 | Whole eggs | USA:MN | 2012 | 4.70 | 0.49 | 30 | 135 |
| CFSAN025717 | Whole eggs | USA:AL | 2012 | 4.70 | 0.30 | 38 | 121 |
| CFSAN025708 | Whole eggs | USA:NJ | 2012 | 4.70 | 0.25 | 43 | 99 |
| CFSAN024814 | Whole eggs | USA:NH | 2012 | 4.69 | 0.31 | 37 | 107 |
| CFSAN024727 | Poultry egg | Chile | 2009 | 4.70 | 0.28 | 41 | 104 |
| CFSAN024743 | Poultry egg | Chile | 2012 | 4.75 | 0.24 | 50 | 102 |
| CFSAN017081 | Frozen liquid egg | USA:GA | 2011 | 4.73 | 0.44 | 35 | 102 |
| CFSAN057651 | Cooked quail eggs | Brazil | 1995 | 4.70 | 0.37 | 38 | 181 |
| CFSAN002042 | Egg slurry | N/A*^b^* | N/A | 4.77 | 0.28 | 35 | 66 |
| CFSAN057762 | Pecked eggs | Brazil | 2004 | 4.69 | 0.27 | 81 | 134 |
| CFSAN057760 | Pecked eggs | Brazil | 2004 | 4.74 | 0.27 | 51 | 128 |
| CFSAN057767 | Pecked eggs | Brazil | 2005 | 4.70 | 0.30 | 42 | 121 |
| CFSAN057769 | Pecked eggs | Brazil | 2005 | 4.64 | 0.31 | 41 | 118 |
| CFSAN057773 | Pecked eggs | Brazil | 2005 | 4.69 | 0.18 | 49 | 91 |
| CFSAN057775 | Pecked eggs | Brazil | 2006 | 4.70 | 0.18 | 47 | 152 |
| CFSAN057780 | Pecked eggs | Brazil | 2006 | 4.70 | 0.18 | 53 | 127 |
| CFSAN057783 | Pecked eggs | Brazil | 2006 | 4.70 | 0.27 | 44 | 234 |
| CFSAN057789 | Pecked eggs | Brazil | 2007 | 4.70 | 0.25 | 52 | 152 |
| CFSAN057791 | Pecked eggs | Brazil | 2007 | 4.70 | 0.24 | 43 | 135 |
| CFSAN057793 | Pecked eggs | Brazil | 2007 | 4.70 | 0.27 | 48 | 124 |
| CFSAN057794 | Pecked eggs | Brazil | 2008 | 4.74 | 0.25 | 89 | 122 |
| CFSAN057795 | Pecked eggs | Brazil | 2008 | 4.70 | 0.25 | 45 | 83 |
| CFSAN057796 | Pecked eggs | Brazil | 2008 | 4.70 | 0.36 | 39 | 433 |
| CFSAN057812 | Pecked eggs | Brazil | 2009 | 4.70 | 0.31 | 45 | 254 |
| CFSAN032971 | Raw egg whites | USA:IA | 2012 | 4.70 | 0.05 | 196 | 34 |
| CFSAN028530 | Raw egg whites | USA:NY | 2012 | 4.70 | 0.38 | 29 | 108 |
| CFSAN030835 | Raw egg whites | USA:CA | 2012 | 4.70 | 0.25 | 41 | 58 |
| CFSAN033541 | Raw egg whites | USA:PA | 2013 | 4.72 | 0.04 | 267 | 84 |
| CFSAN027377 | Raw egg yolks | USA:NJ | 2012 | 4.75 | 0.15 | 110 | 40 |
| CFSAN030066 | Raw egg yolks | USA:NY | 2012 | 4.70 | 0.20 | 51 | 46 |
| CFSAN030067 | Raw egg yolks | USA:IA | 2012 | 4.69 | 0.25 | 36 | 62 |
| CFSAN030097 | Raw egg yolks | USA:IN | 2012 | 4.70 | 0.24 | 39 | 93 |
| CFSAN030496 | Raw egg yolks | USA:GA | 2012 | 4.69 | 0.11 | 101 | 70 |
| CFSAN030816 | Raw egg yolks | USA:NJ | 2012 | 4.73 | 0.17 | 56 | 75 |
| CFSAN030839 | Raw egg yolks | USA:WA | 2012 | 4.69 | 0.28 | 41 | 58 |
| CFSAN030852 | Raw egg yolks | USA:NY | 2012 | 4.73 | 0.27 | 51 | 29 |
| CFSAN032958 | Raw egg yolks | USA:NJ | 2012 | 4.70 | 0.25 | 41 | 67 |
| CFSAN032959 | Raw egg yolks | USA:NJ | 2012 | 4.68 | 0.11 | 92 | 57 |
| CFSAN032964 | Raw egg yolks | USA:NY | 2012 | 4.69 | 0.10 | 91 | 44 |
| CFSAN032970 | Raw egg yolks | USA:IA | 2012 | 4.72 | 0.13 | 138 | 76 |
| CFSAN035289 | Raw egg yolks | USA:NJ | 2012 | 4.70 | 0.30 | 39 | 55 |
| CFSAN035308 | Raw egg yolks | USA:AL | 2012 | 4.70 | 0.27 | 48 | 56 |
| CFSAN035309 | Raw egg yolks | USA:IA | 2012 | 4.70 | 0.29 | 34 | 101 |
| CFSAN034151 | Raw egg yolks | USA:GA | 2013 | 4.71 | 0.08 | 124 | 36 |
| CFSAN027378 | Raw whole eggs | USA:UT | 2012 | 4.74 | 0.21 | 68 | 67 |
| CFSAN027394 | Raw whole eggs | USA:NH | 2012 | 4.70 | 0.28 | 40 | 93 |
| CFSAN030081 | Raw whole eggs | USA:NJ | 2012 | 4.70 | 0.31 | 40 | 66 |
| CFSAN030086 | Raw whole eggs | USA:IA | 2012 | 4.63 | 0.21 | 68 | 54 |
| CFSAN030823 | Raw whole eggs | USA:OH | 2012 | 4.73 | 0.29 | 71 | 54 |
| CFSAN032962 | Raw whole eggs | USA:IA | 2012 | 4.71 | 0.04 | 225 | 25 |
| CFSAN033543 | Raw whole eggs | USA:OH | 2012 | 4.69 | 0.15 | 66 | 82 |
| CFSAN035276 | Raw whole eggs | USA:NY | 2012 | 4.71 | 0.29 | 40 | 84 |
| CFSAN035291 | Raw whole eggs | USA:NJ | 2012 | 4.71 | 0.26 | 55 | 56 |
| CFSAN034231 | Raw whole eggs | USA:GA | 2013 | 4.69 | 0.25 | 35 | 79 |
| CFSAN034232 | Raw whole eggs | USA:GA | 2013 | 4.70 | 0.33 | 31 | 116 |
| CFSAN035272 | Raw whole eggs | USA:TX | 2013 | 4.73 | 0.34 | 44 | 89 |
| CFSAN057837 | Chicken | Brazil | 1990 | 4.74 | 0.26 | 43 | 230 |
| CFSAN057838 | Chicken | Brazil | 1990 | 4.75 | 0.19 | 62 | 253 |
| CFSAN057841 | Chicken | Brazil | 1993 | 4.69 | 0.19 | 48 | 269 |
| CFSAN057814 | Chicken | Brazil | 2010 | 4.79 | 0.23 | 66 | 354 |
| CFSAN058030 | Chicken | USA:NJ | 2014 | 4.74 | 0.22 | 49 | 45 |

*^a^* AL, Alabama; CA, California; GA, Georgia; IA, Iowa; IN, Indiana; MN, Minnesota; NH, New Hampshire; NJ, New Jersey; NY, New York; OH, Ohio; PA, Pennsylvania; TX, Texas; UT, Utah; WA, Washington.

*^b^* No information in the database.

**Supplementary Table 2. Sources of each *Salmonella* ser. Typhimurium isolate and their *de novo* assembled genomes.**

| **Isolates** | **Source** | **Location***^a^* | **Year** | **Genome**  **Size (Mb)** | **N50 (Mb)** | **# of contigs** | **Depth of coverage** |
| --- | --- | --- | --- | --- | --- | --- | --- |
|  |  |  |  |  |  |  |  |
| CFSAN017093 | Duck egg yolks (Cooked, Frozen) | China | 2010 | 4.90 | 0.15 | 80 | 62 |
| CFSAN017094 | Duck egg yolks (Cooked, Frozen) | China | 2010 | 4.91 | 0.22 | 75 | 105 |
| CFSAN017095 | Duck egg yolks (Cooked, Frozen) | China | 2010 | 4.94 | 0.28 | 69 | 121 |
| CFSAN015377 | Frozen salted duck yolk | China | 2002 | 4.89 | 0.22 | 55 | 81 |
| CFSAN015378 | Frozen salted duck yolk | China | 2002 | 5.07 | 0.38 | 63 | 82 |
| CFSAN015380 | Frozen salted duck yolk | China | 2002 | 4.89 | 0.22 | 64 | 136 |
| CFSAN013737 | Salted egg yolk | China (Taiwan) | 2001 | 4.92 | 0.30 | 49 | 91 |
| CFSAN014205 | Salted duck eggs | China (Taiwan) | 2004 | 4.91 | 0.18 | 54 | 80 |
| CFSAN015282 | Chicken jerky | China | 2001 | 5.01 | 0.20 | 72 | 75 |
| CFSAN027862 | Chicken breast | USA:CO | 2005 | 5.02 | 0.08 | 282 | 62 |
| CFSAN029083*^a^* | Chicken breast | USA:GA | 2006 | 5.09 | 0.09 | 144 | 71 |
| CFSAN029101 | Chicken breast | USA:MD | 2006 | 5.07 | 0.11 | 101 | 75 |
| CFSAN029123 | Chicken breast | USA:MD | 2006 | 5.11 | 0.22 | 65 | 88 |
| CFSAN035417 | Chicken breast | USA:CA | 2007 | 4.89 | 0.19 | 54 | 83 |
| CFSAN035525 *^a^* | Chicken breast | USA:CT | 2007 | 5.11 | 0.18 | 79 | 90 |
| CFSAN035560 | Chicken breast | USA:MD | 2008 | 4.98 | 0.19 | 83 | 87 |
| CFSAN035575 | Chicken breast | USA:NY | 2008 | 5.07 | 0.17 | 85 | 49 |
| CFSAN036172 *^a^* | Chicken breast | USA:MD | 2008 | 5.01 | 0.21 | 58 | 146 |
| CFSAN036174 *^a^* | Chicken breast | USA:NM | 2008 | 4.85 | 0.13 | 89 | 94 |
| CFSAN036177 *^a^* | Chicken breast | USA:NY | 2008 | 5.08 | 0.22 | 71 | 163 |
| CFSAN036179 *^a^* | Chicken breast | USA:NY | 2008 | 4.97 | 0.15 | 66 | 107 |
| CFSAN036183 *^a^* | Chicken breast | USA:PA | 2008 | 5.19 | 0.25 | 73 | 94 |
| CFSAN036186 *^a^* | Chicken breast | USA:PA | 2008 | 4.97 | 0.29 | 55 | 149 |
| CFSAN036257 *^a^* | Chicken breast | USA:MD | 2008 | 5.02 | 0.24 | 60 | 117 |
| CFSAN036272 *^a^* | Chicken breast | USA:MD | 2008 | 5.02 | 0.27 | 65 | 101 |
| CFSAN036362 *^a^* | Chicken breast | USA:NY | 2008 | 5.01 | 0.26 | 61 | 126 |
| CFSAN036367 *^a^* | Chicken breast | USA:NY | 2008 | 5.00 | 0.27 | 56 | 73 |
| CFSAN041824 *^b^* | Chicken breast | USA:NY | 2009 | 4.97 | 0.22 | 56 | 118 |
| CFSAN041835 *^b^* | Chicken breast | USA:NY | 2009 | 5.00 | 0.19 | 79 | 87 |
| CFSAN041875 *^b^* | Chicken breast | USA:PA | 2009 | 5.00 | 0.22 | 78 | 174 |
| CFSAN041878 *^b^* | Chicken breast | USA:PA | 2009 | 5.07 | 0.28 | 64 | 100 |
| CFSAN041887 *^b^* | Chicken breast | USA:PA | 2009 | 5.09 | 0.11 | 117 | 71 |
| CFSAN040229 | Chicken breast | USA:MD | 2009 | 4.95 | 0.09 | 109 | 92 |
| CFSAN041662 *^b^* | Chicken breast | USA:TN | 2010 | 4.96 | 0.16 | 74 | 86 |
| CFSAN041925 | Chicken breast | USA:CT | 2010 | 4.98 | 0.18 | 75 | 101 |
| CFSAN041934 *^b^* | Chicken breast | USA:GA | 2010 | 4.95 | 0.23 | 60 | 57 |
| CFSAN041940 *^b^* | Chicken breast | USA:MD | 2010 | 4.98 | 0.10 | 109 | 65 |
| CFSAN041947 *^b^* | Chicken breast | USA:MD | 2010 | 4.96 | 0.28 | 60 | 93 |
| CFSAN041965 *^b^* | Chicken breast | USA:MN | 2010 | 4.94 | 0.21 | 79 | 103 |
| CFSAN041987 *^b^* | Chicken breast | USA:NY | 2010 | 4.99 | 0.10 | 104 | 55 |

*^a^* CA, California; CT, Connecticut; CO, Colorado; GA, Georgia; MD, Maryland; MN, Minnestota; NM, New Mexico; NY, New York; PA, Pensylvania; TN, Tennessee.

*^a^* Typhimurium var. 5-.

*^b^* Typhimurium var. O:5-.

**Supplementary Table 3. Sources of each *Salmonella* ser. Heidelberg isolate and their *de novo* assembled genomes.**

| **Isolates** | **Source** | **Location***^a^* | **Year** | **Genome**  **Size (Mb)** | **N50 (Mb)** | **# of contigs** | **Depth of coverage** |
| --- | --- | --- | --- | --- | --- | --- | --- |
|  |  |  |  |  |  |  |  |
| CFSAN015479 | Egg salad | USA:WA | 2003 | 4.75 | 0.69 | 29 | 77 |
| CFSAN024803 | Egg yolks | USA:IA | 2012 | 4.79 | 0.29 | 32 | 44 |
| CFSAN024816 | Egg yolks | USA:IA | 2012 | 4.75 | 0.30 | 33 | 132 |
| CFSAN024838 | Egg yolks | USA:IA | 2012 | 4.81 | 0.23 | 39 | 86 |
| CFSAN025710 | Egg yolks | USA:IN | 2012 | 4.75 | 0.38 | 28 | 127 |
| CFSAN028512 | Raw eggs whites | USA:IA | 2012 | 4.77 | 0.06 | 159 | 38 |
| CFSAN033551 | Raw eggs whites | USA:NJ | 2012 | 4.77 | 0.23 | 44 | 62 |
| CFSAN033559 | Raw eggs whites | USA:IA | 2013 | 4.79 | 0.15 | 56 | 55 |
| CFSAN035286 | Raw eggs whites | USA:IA | 2013 | 4.79 | 0.41 | 40 | 63 |
| CFSAN027390 | Raw eggs yolks | USA:IA | 2012 | 4.79 | 0.38 | 35 | 61 |
| CFSAN028528 | Raw eggs yolks | USA:IA | 2012 | 4.83 | 0.23 | 49 | 36 |
| CFSAN032955 | Raw eggs yolks | USA:AL | 2012 | 4.75 | 0.20 | 41 | 73 |
| CFSAN035301 | Raw eggs yolks | USA:IN | 2012 | 4.81 | 0.25 | 42 | 85 |
| CFSAN033560 | Raw eggs yolks | USA:IA | 2013 | 4.77 | 0.25 | 42 | 50 |
| CFSAN035287 | Raw eggs yolks | USA:AL | 2013 | 4.80 | 0.17 | 62 | 52 |
| CFSAN027397 | Raw whole eggs | USA:SC | 2012 | 4.75 | 0.29 | 32 | 68 |
| CFSAN028516 | Raw whole eggs | USA:AL | 2012 | 4.75 | 0.27 | 36 | 33 |
| CFSAN033536 | Raw whole eggs | USA:MN | 2012 | 4.78 | 0.16 | 46 | 47 |
| CFSAN033547 | Raw whole eggs | USA:GA | 2012 | 4.74 | 0.23 | 48 | 71 |
| CFSAN035307 | Raw whole eggs | USA:IA | 2012 | 4.74 | 0.25 | 39 | 66 |
| CFSAN034130 | Raw whole eggs | USA:MN | 2012 | 4.79 | 0.19 | 50 | 77 |
| CFSAN034209 | Raw whole eggs | USA:AL | 2013 | 4.76 | 0.02 | 473 | 43 |
| CFSAN033552 | Raw whole eggs | USA:IN | 2013 | 4.72 | 0.11 | 106 | 26 |
| CFSAN033555 | Raw whole eggs | USA:GA | 2013 | 4.78 | 0.30 | 39 | 48 |
| CFSAN034223 | Raw whole eggs | USA:NJ | 2013 | 4.74 | 0.02 | 446 | 73 |
| CFSAN035285 | Raw whole eggs | USA:IN | 2013 | 4.74 | 0.30 | 31 | 82 |
| CFSAN024808 | Whole eggs | USA:SC | 2012 | 4.75 | 0.30 | 34 | 112 |
| CFSAN024821 | Whole eggs | USA:MI | 2012 | 4.79 | 0.44 | 33 | 134 |
| CFSAN024830 | Whole eggs | USA:NJ | 2012 | 4.77 | 0.28 | 34 | 149 |
| CFSAN025697 | Whole eggs | USA:AL | 2012 | 4.81 | 0.30 | 33 | 217 |
| CFSAN035462 | Chicken breast | USA:CO | 2007 | 4.74 | 0.30 | 34 | 78 |
| CFSAN035479 | Chicken breast | USA:NM | 2007 | 4.76 | 0.24 | 40 | 145 |
| CFSAN035554 | Chicken breast | USA:CA | 2008 | 5.06 | 0.30 | 40 | 123 |
| CFSAN036211 | Chicken breast | USA:CT | 2008 | 4.90 | 0.20 | 54 | 110 |
| CFSAN036243 | Chicken breast | USA:GA | 2008 | 4.77 | 0.29 | 39 | 111 |
| CFSAN036278 | Chicken breast | USA:MN | 2008 | 4.85 | 0.32 | 33 | 132 |
| CFSAN036283 | Chicken breast | USA:NM | 2008 | 5.03 | 0.42 | 39 | 151 |
| CFSAN036285 | Chicken breast | USA:NM | 2008 | 4.75 | 0.16 | 61 | 118 |
| CFSAN041699 | Chicken breast | USA:CA | 2010 | 5.03 | 0.15 | 74 | 76 |

*^a^* AL, Alabama; CA, California; CO, Colorado; CT, Connecticut; GA, Georgia; IA, Iowa; IN, Indiana; MI, Michigan; MN, Minnesota; NM, New Mexico; NJ, New Jersey; SC, South Carolina; WA, Washington.

**
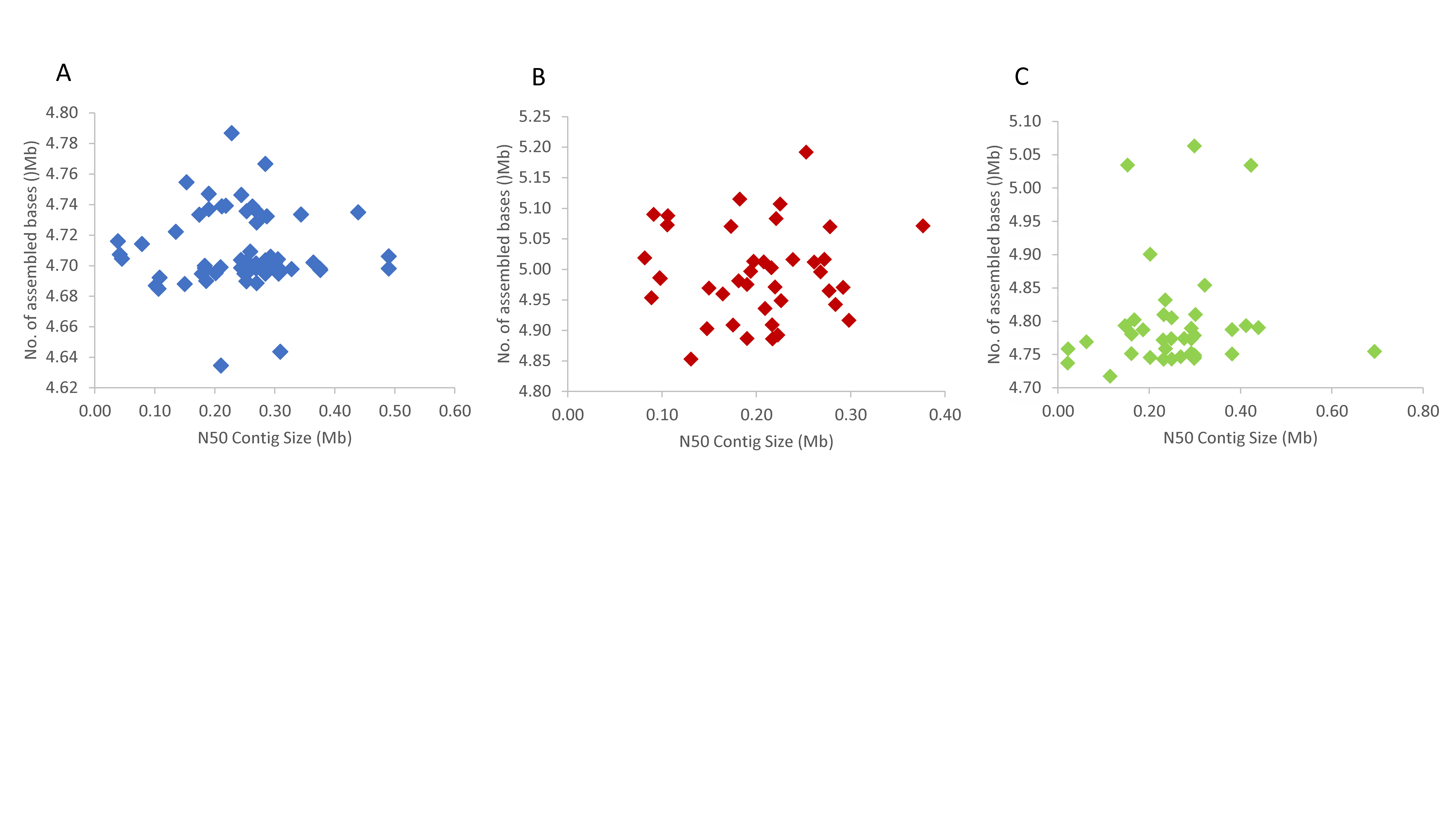
**

**Supplementary Figure 1.** Number of assembled bases (Mb) and N50 contig sizes (Mb) for sequenced *Salmonella* ser. Enteritidis (A), *Salmonella* ser. Typhimurium (B), and *Salmonella* ser. Heidelberg (C) isolates.
